# The rs6771157 C/G polymorphism in *SCN10A* is associated with the risk of atrial fibrillation in a Chinese Han population

**DOI:** 10.1038/srep35212

**Published:** 2016-10-11

**Authors:** Zhen Fang, Yue Jiang, Yifeng Wang, Yuan Lin, Yaowu Liu, Liyan Zhao, Yan Xu, Mohammad Bilaal Toorabally, Shenghu He, Fengxiang Zhang

**Affiliations:** 1 Department of Cardiology, Clinical Medical College, Yangzhou University, Yangzhou, Jiangsu, China; 2 Department of Cardiology, The First Affiliated Hospital of Nanjing Medical University, Nanjing, Jiangsu, China; 3 Department of Epidemiology and Biostatistics, School of Public Health, Nanjing Medical University, Nanjing, Jiangsu, China; 4 Department of Cardiology, Zhongda Hospital of Southeast University, Nanjing, Jiangsu, China

## Abstract

A recent genome wide associated study in European descent population identified the association of Atrial fibrillation (AF) risk with a single nucleotide polymorphism (SNP) in *SCN10A*. The aim of this study was to evaluate whether *SCN10A* polymorphisms are associated with AF risk in the Chinese Han population. A total of 2,300 individuals of Chinese Han origin were recruited and three potentially functional SNPs were genotyped. Logistic regression models were utilized to calculate odds ratios (ORs) at a 95% confidence intervals (CIs). Logistic regression analysis in an additive genetic model revealed that one SNP in *SCN10A* (rs6771157) was associated with an increased risk of AF (adjusted OR = 1.20, 95% CI: 1.06 - 1.36, *P *= 0.003). Stratification analysis of several main AF risk factors indicated that the risk associations with rs6771157 were not statistically different among different subgroups. In summary, our study suggests the possible involvement of the *SCN10A* variant in AF development in Chinese Han populations. Further biological function analyses are required to confirm our finding.

Atrial fibrillation (AF), one of the most prevalent arrhythmia in clinical practice, affects 1–2% of the general population^1^. AF is related to a five-fold increased risk of stroke^2^, a three-fold increase in incidence of congestive heart failure^3^ and a two-fold increase in mortality rate^4^,^5^, which contribute to higher hospitalization rate in AF patients^3^.

The occurrence of AF is usually considered to be associated with multiple cardiovascular risk factors, such as advanced age, male, hypertension, diabetes, heart failure, and hyperthyroidism^4^,^6^,^7^. However, a small part of the patients with AF lack the clinical evidence of risk factors. We named this type of AF as ‘lone AF’, which account for 10–20% of the total AF^8^. Several studies have demonstrated that AF, especially lone AF, have a predisposing genetic component^9^,^10^,^11^,^12^. Over the past decade, multiple genetic variants, including common and rare genetic variants, had been reported to be associated with susceptibility to AF^13^,^14^,^15^. In 2010, a genome-wide association study (GWAS) by Pfeufer *et al*. detected an intronic SNP (rs6800541) in *SCN10A* that is independently associated with PR-interval and AF risk (*P *= 9.7 × 10^−82^, *P* = 0.5 × 10^−4^, respectively)^16^. A meta-analysis of 14 GWASs discovered that another intronic SNP (rs6801957) in *SCN10A* may be a major risk marker for prolonged QRS duration in individuals of European descent^17^. Further functional analyses have confirmed that *SCN10A* was expressed in human ventricular conduction system and the loss of *SCN10A* had an apparent impact on both PR interval and QRS duration in mouse models^17^,^18^. These facts indicate that *SCN10A* may be involved in the development of AF. In this study, we employed candidate gene approach to identify potentially functional AF susceptibility SNPs in *SCN10A* in Chinese Han populations.

## Results

### Characteristics of the Study Population

We recruited 2,300 individuals of Chinese Han origin in this study totally, including 1,150 AF cases and 1,150 AF-free controls. The clinical characteristics between cases and controls are summarized in Table 1. In brief, the variants of gender and age in two groups were comparable (*P *> 0.05). Compared with the non-AF subjects, AF patients were more likely to have the percentages of hypertension, diabetes and coronary artery disease (CAD) (45.1% vs. 20.8%, *P *< 0.001 for hypertension, 10.5% vs. 7.5%, *P* = 0.011 for diabetes and 10.3% vs. 0%, *P *< 0.001 for CAD, respectively).

### Associations between *SCN10A* variants and AF risk

The genotype distributions of the three SNPs and their associations with AF risk are shown in Table 2. The genotype frequencies of these SNPs were all observed in agreement with Hardy–Weinberg equilibrium in the control group. The logistic regression analyses revealed that the rs6771157 C allele was significantly associated with the increased risk of AF in an additive model (adjusted OR = 1.20, 95% CI: 1.06–1.36, *P* = 0.003). We further calculated *P* values for false discovery rate (*P*-FDR) to perform multiple comparisons, which found that rs6771157 still remained association with the risk of AF (*P*-FDR = 0.010). There was no obvious evidence of significant association between the other two SNPs and AF risk.

### Stratification Analysis

We assessed the effect size of rs6771157 with AF by stratification analyses based on the main AF risk factors such as age, gender, hypertension, diabetes. As shown in Table 3, no significant difference for the association of rs6771157 with AF risk between subgroups was detected (*P *> 0.05 for heterogeneity test).

## Discussion

This case-control study investigated the relationship between the common genetic variants of *SCN10A* and their potential function in the risk of AF in the Chinese Han population. Only SNP rs6771157 was identified to be significantly associated with Chinese Han AF risk.

*SCN10A* encodes the voltage-gated sodium channel, Nav1.8 channels. The protein product of *SCN10A* is predominant tetrodotoxin-resistant sodium channel in primary sensory neurons^19^,^20^. Although the exact role of Nav1.8 in cardiac electrophysiology remains currently unclear^21^, *SCN10A* is expressed both in intracardiac neurons and human cardiomyocytes^22^,^23^. Further, blockade of Nav.18 channels suppresses the impact of vagus nerve stimulation (VNS) on both cardiac conduction and AF inducibility^23^,^24^,^25^. Thus, *SCN10A* plays an important role in cardiac electrophysiology and modulating susceptibility to arrhythmias^24^.

**Figure 1:**
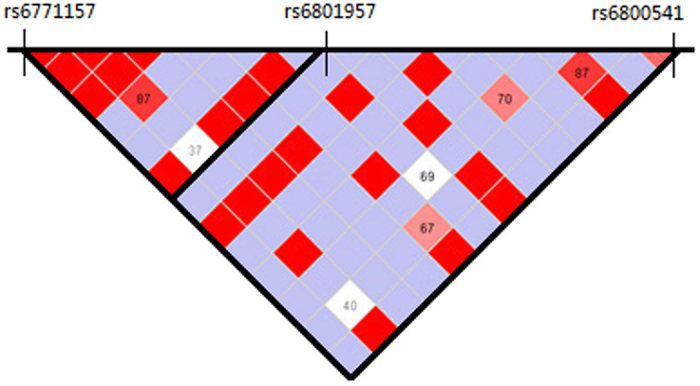
A regional plot of *SCN10A* gene to characterize the association results and linkage disequilibrium (LD) of the three SNPs. The pairwise correlation between the SNP rs6771157 and two SNPs (rs6800541, rs6801957) were measured with r^2^, r^2^ = 0.1 and 0.171 respectively.

In a previous GWAS. A tag SNP (rs6800541) in *SCN10A* has been found to have a strong association with PR-interval duration and AF^16^. Most recently, a function study by Jabbari *et al*. revealed two common nonsynonymous variants in *SCN10A* (rs6795970, rs12632942) result in a gain-of-function of Nav1.8 channel^21^. In the present study, we found an association between another common variant rs6771157 and AF risk in Chinese Han populations. We analyzed the linkage disequilibrium of SNP rs6771157 with the two significant SNPs via haploview software, which showed r^2^ = 0.1 and 0.171 for rs6800541 and rs6801957 respectively (Fig. 1). Thus, the rs6771157, a synonymous variant located in the 19 exon of *SCN10A*. is a signal different from the two SNPs reported in European individuals. It was predicted as a functional SNP in an ESE (exon splicing enhancer) by the online tool SNPinfo (http://snpinfo.niehs.nih.gov/index.html), which may be responsible for aberrant splicing of pre-mRNA of *SCN10A* by binding to the SR proteins of splicing activators^26^. A possible explanation is that rs6771157 causes the exon skipping by activating the creation of the ESE.

Several limitations of the present study need to be considered. Firstly, the sample size is relatively small compared to GWAS, which may predispose to the failure to detect effects of another two potential functional SNPs in our study. Secondly, echocardiography results and N-terminal of the prohormone brain natriuretic peptide (NT-proBNP) levels were not available for all the individuals to evaluate cardiac functions, which may have resulted in information bias. We did not evaluate cardiac functions by left ventricular ejection fraction (LVEF) and NT-proBNP. Furthermore, we selected the potential functional SNP by using on-line tools, which may result in positive or negative errors. Thus, further replication in larger sample sets and mechanism researches are required to confirm our findings.

In conclusion, the current investigation confirmed that one functional common variation (rs6771157) in *SCN10A* was significantly associated with the risk of AF in a Chinese Han population. However, the role of this variant in the AF susceptibility is warranted to be further evaluated by function studies.

## Methods

### Study population

This study was approved by Ethical Committee Review Board of Nanjing Medical University, China. The written informed consents were obtained from all the participants enrolled in the study. The experimental protocol was carried out in accordance with the approved guidelines.

The AF cases were recruited from Department of Cardiology, the First Affiliated Hospital of Nanjing Medical University from June 2010 to December 2014. We included AF patients according to their routine 12-lead electrocardiography (ECG), or holter ECG recordings which were characterized with irregular R-R intervals, absence of distinct repeating P waves, and irregular atrial activity^27^. We classified AF as paroxysmal AF (terminates spontaneously or with intervention within 7 days of onset), persistent AF (episodes that sustain beyond 7 days). We defined “Lone AF” as young AF individuals (<60 years) without clinical or echocardiographic evidence of cardiopulmonary disease^3^. The controls were recruited from other departments of the First Affiliated Hospital of Nanjing Medical University, and they were all confirmed to be free of AF based on medical files or ECG at the time of enrollment.

The general and clinical information of all participants was collected from medical recording files in the hospital system. We excluded patients with hyperthyroidism, severe cardiac dysfunction (NYHA Class IV), valvular heart disease and advanced age (beyond 90 years) in AF group. All individuals were Chinese Han origin.

### SNP selection

We first used public HapMap SNP database (phase II+III Feb 09, on NCBI B36 assembly, dbSNP b126) to search SNPs that localized within the gene region of *SCN10A* (including 10 kb upstream of the gene), with MAF (Minor Allele Frequency) ≥ 0.05 in Chinese Han population. Then, a web-based analysis tool was used to predict the function of these SNPs (http://snpinfo.niehs.nih.gov/snpinfo/snpfunc.htm). After function prediction analysis, a total of six potentially functional SNPs were selected. We conducted linkage disequilibrium (LD) analysis to exclude SNPs with strong LD (r^2^ ≥ 0.8), then three (rs9827941, rs7630989, and rs6771157) functional SNPs were selected for further genotyping.

### SNP genotyping

Peripheral venous blood samples were drawn from study participants. Genomic DNA was extracted from EDTA-preserved whole blood using a standard phenol-chloroform method^28^. The genotyping was conducted using the method of the improved multiple ligase detection reaction (iMLDR) with the technical assistance from Shanghai Genesky Bio-Tech Genetic Core Lab. All SNPs were genotyped successfully with call rates at least of 99.3%.

### Statistical analysis

The comparisons of clinical characteristic differences between cases and controls were identified using Student’s *t* test for continuous variables and the *χ*^2^ test for categorical variables. The deviation of genotype distribution from Hardy-Weinberg equilibrium was tested by *χ*^2^ test in the control group. ORs and 95% CIs were calculated to assess the relationships between the SNPs and the risk of AF using logistic regression. The heterogeneity of associations among subgroups was evaluated using the *χ*^2^-based *Q*-test. All statistical analyses were performed with STATA 12.0 software (Stata Corp., College Station, TX, USA). *P *< 0.05 was the criterion of statistical significance and all statistical tests were two-tailed. LD analysis was applied by using Haploview 4.2 software.

## Additional Information

**How to cite this article**: Fang, Z. *et al*. The rs6771157 C/G polymorphism in *SCN10A* is associated with the risk of atrial fibrillation in a Chinese Han population. *Sci. Rep.* **6**, 35212; doi: 10.1038/srep35212 (2016).

## Figures and Tables

**Table 1:** Clinical characteristics of the Chinese Han study population.

Variants	Cases	Controls	*P* value
	(N = 150)	(N = 150)	
Male gender (%)	757(65.8%)	757(65.8%)	1
Age, years	58.7 ± 11.5	59.1 ± 10.5	0.379
Paroxysmal AF (%)	559 (48.6%)	NA	—
Persistent AF (%)	591(51.4%)	NA	—
Lone AF (%)	152(13.2%)	NA	—
Hypertension (%)	519 (45.1%)	239 (20.8%)	<0.001
Diabetes (%)	121 (10.5%)	86 (7.5%)	0.011
CAD (%)	118 (10.3%)	0 (0.0%)	<0.001

AF, atrial fibrillation; CAD, coronary artery disease; NA, not available.

**Table 2:** Summary of associations between 3 SNPs in *SCN10A* and the risk of AF.

SNP	Position	Minor/major	Genotype distribution^a^			MAF		Crude ES^b^		Adjusted ES^c^		
			Cases	Controls	Cases	Controls	*P*_HWE_	OR(95% CI)	*P* Value	OR(95% CI)	*P* Value	*P*_FDR_^d^
rs9827941	38811463	A/T	80/444/626	87/425/622	0.26	0.26	0.23	0.99(0.87–1.13)	0.909	1.01(0.88–1.16)	0.910	0.910
rs7630989	38768944	G/A	63/360/727	59/371/720	0.21	0.21	0.22	0.99(0.87–1.14)	0.916	1.03(0.89–1.19)	0.741	0.910
rs6771157	38738867	C/G	255/581/314	217/535/398	0.47	0.42	0.12	**1.24(1.10–1.39)**	**3.48** × **10**^**−4**^	**1.20(1.06–1.36)**	**0.003**	**0.010**

SNP, single nucleotide polymorphism; MAF, minor allele frequency; *P*_HWE_, *P* values for Hardy–Weinberg equilibrium tests in the control group; ES effect size; OR odds ratio; CI confidence interval.

^a^Genotype distribution for the minor allele/heterozygous/homozygous.

^b^ES were derived from logistic regression analysis in the additive model for unadjustment of any covariants.

^c^ES were derived from logistic regression analysis in the additive model for adjustment of age, gender, hypertension, diabetes and coronary artery disease.

^d^Multiple comparisons *P* values for false discovery rate.

**Table 3:** Stratified analysis on the associations of SNP in *SCN10A* with AF.

Variables	rs6771157	(CC/CG/GG)	Adjusted OR^a^ (95% CI)	*P*^b^
	Cases	Controls		
**Age**^c^				
≤59	129/284/160	99/271/197	1.24 (1.05–1.47)	0.850
≥60	126/297/154	118/264/201	1.21 (1.00–1.46)	
**Gender**				
male	160/391/206	150/344/263	1.18 (1.01–1.37)	0.576
female	95/190/108	67/191/135	1.27 (1.03–1.56)	
**Diabetes**				
Yes	26/68/27	15/38/33	1.56 (0.98–2.48)	0.256
No	229/513/287	202/497/365	1.18 (1.04–1.34)	
**Hypertension**				
Yes	112/266/141	48/100/91	1.34 (1.06–1.68)	0.366
No	143/315/173	169/435/307	1.18 (1.02–1.38)	

^a^Obtained in logistic regression models with adjustment for age, gender, hypertension, diabetes and coronary artery disease (the stratified factor in each stratum excluded).

^b^*P* for heterogeneity test using the Chi-square-based *Q* test.

^c^Age was divided into two subgroups according to its median (59 years).
